# Breast Milk Macronutrient Components in Prolonged Lactation

**DOI:** 10.3390/nu10121893

**Published:** 2018-12-03

**Authors:** Matylda Czosnykowska-Łukacka, Barbara Królak-Olejnik, Magdalena Orczyk-Pawiłowicz

**Affiliations:** 1 Neonatology Department, Wroclaw Medical University, Borowska 213, 50-556 Wroclaw, Poland; barbara.krolak-olejnik@umed.wroc.pl; 2 Department of Chemistry and Immunochemistry, Wroclaw Medical University, Bujwida 44a, 50-345 Wroclaw, Poland; magdalena.orczyk-pawilowicz@umed.wroc.pl

**Keywords:** breastfeeding, macronutrients, prolonged lactation, child nutrition

## Abstract

Human milk (HM) is the first dietary exposure in infancy and the best nutritional option for growth and healthy development of the newborn and infant. The concentrations of macronutrients, namely proteins, carbohydrates and lipids, change during lactation stages. HM has been studied extensively in the first 6 months of lactation, but there are limited data regarding milk composition beyond 1 or 2 years postpartum. The aim of our study was to describe longitudinal changes in HM macronutrient concentrations during the prolonged lactation of healthy mothers from the 1st to the 48th month. For the macronutrient content of milk of mothers breastfeeding for longer than 18 months, fat and protein increased and carbohydrates decreased significantly, compared with milk expressed by women breastfeeding up to 12 months. Moreover, the concentration of fat, protein and carbohydrates in HM over 2 years of lactation from the 24th to the 48th month remained at a stable level. However, analyzed macronutrients were positively (carbohydrate, *r* = 0.51) or negatively (fat, *r* = −0.36 and protein, *r* = −0.58) correlated with the amount of feeding. Our results create a greater potential for understanding the nutritional contribution of HM over 2 years of lactation and showed that the source of calories in breast milk for older children is mainly fat, while carbohydrates play a greater role in infant nutrition in the early stage. The observed changes of macronutrient concentrations from the 1st to the 48th month of lactation are probably related to the adaptation of milk composition to the increased energy demand of the intensively growing child.

## 1. Introduction

### 1.1. Importance of Breastfeeding

Breastfeeding and human milk are the gold standard for infant feeding and nutrition [1]. The World Health Organization (WHO) recommends exclusive breastfeeding for the first 6 months, alongside continued breastfeeding up to the second year and beyond [1]. The American Academy of Pediatrics (AAP) recommends exclusive breastfeeding for about 6 months, followed by continued breastfeeding as complementary foods are introduced, with continuation of breastfeeding for 1 year or longer as mutually desired by mother and infant [2]. Unfortunately, the WHO reported that only 36% of infants worldwide were exclusively breastfed until the age of 6 months’ from 2007 to 2014 [3,4]. In addition, maternal concerns about insufficient milk supply and nursing discomfort in the first week postpartum are associated with early cessation of breastfeeding [5,6,7,8]. There is a tendency to think that this is a relatively recent trend associated with the development of infant formula, but across recorded history there are descriptions of the use of wet nurses and of animal milk substitutes to feed babies [9,10].

Human milk is the first and the best feeding option for growth and healthy development of the newborns and infants [11]. Human milk contains numerous components (i.e., proteins, carbohydrates, lipids, and inorganic elements) which provide basic nutrients for infants during the first period of their lives. The qualitative composition of milk components from healthy mothers is similar, but their levels change during lactation stages [12]. Colostrum is the fluid secreted during the first days postpartum by mammary epithelial cells. Colostrum is replaced by transitional milk during 5–15 days postpartum, and from 15 days postpartum mature milk is produced. Human milk, apart from the nutritional components, is a source of biologically active molecules, i.e., immunoglobulins, growth factors, hormones, cytokines, acute phase proteins, antiviral, and antibacterial proteins. These bioactive molecules present in the milk support the immature immune system of the newborn and also protect against the development of infection [13,14].

Moreover, in recent years, breast milk has been shown to be a continuous source of bacteria for the infant gut as well, including staphylococci, streptococci, bifidobacterial, and lactobacilli [14,15,16,17,18]. Increasing knowledge of the role of epigenetics, stem cells, and the developmental origins of health and disease also contributes to promoting the benefits of breastfeeding. It needs to be underlined that breastfeeding might permanently shape an individuals’ life course/well-being. Prolonged breastfeeding, however, may have an impact on other factors associated with modulation of the immune system as well as metabolic programming.

### 1.2. Stages of Lactation

Lactation is a dynamic process [19]. The variability of the macronutrient content of human milk is very large. Many factors may affect the volume and composition of milk, the most influential being the stage of lactation, although time of delivery [20] and degree of prematurity also play a role in milk composition [21]. The composition of HM is widely believed to be specifically tailored by each mother to precisely reflect the requirements of her infant [22].

Maternal diet has a slight effect on the amount of nutrients in human milk [23,24]. Maternal nutrition and breast milk fatty acids are correlated with maternal lipid nutrition and the possibility that poor fatty acid nutrition can alter neurological development in breastfed infants [25,26].

Concentration of fats in colostrum and carbohydrates in mature milk from mothers with advanced age are elevated compared with those of younger mothers [27]. Mother’s age may be a factor in the fatty acids composition of human milk. This should be taken into account when planning diets for pregnant women of different ages [28].

Milk composition depends on the length of feeding, time of the day, lactation period, date of delivery, mother and population. Understanding human milk composition provides an important tool for management of infant feeding, especially preterm infants, but also toddlers when mothers breastfeed for more than 1 year. Furthermore, the benefits of breastfeeding for development seem to extend beyond the first 2 years of life. Unfortunately, research on this issue cannot be carried out as randomized trials are affected by confounding variables affecting the supply of mother’s milk and the outcome of the infants [29,30].

Human milk composition has been studied extensively in the first half year after parturition, but there is a paucity of data regarding milk composition beyond 1 and 2 years postpartum [30,31,32]. In particular, the fat and energy contents of HM in long-term breastfeeding have not been analyzed systematically except a few studies have included mothers who had been lactating from 1 to 2 years [27,30]. The Workshop on Human Milk Composition which took place in November 2017 organized by National Institute of Health (NIH) and U.S. Department of Agriculture (USDA) demonstrated gaps in the knowledge about human milk composition. The limitations of the available studies on human milk content are as follows: Small sample sizes at later study time points, women are mainly from western countries and do not reflect inter-individual variations that can occur between mothers’ milk around the world [27,33]. Filling this research gap will lead to a greater understanding of the nutritional contribution of human milk beyond 1 or even 2 and 3 years of lactation.

To the best of our knowledge, there is no scientific data concerning the analysis of human milk macronutrients over 2 years of lactation. Therefore, the primary purpose of this study is to describe longitudinal changes in human milk composition during prolonged lactation. The scope of this research includes: analysis of macronutrients (total protein, total fat and lactose) in human milk up to 12, 18, 24 and above 24 months of lactation. Mothers are often informed that milk after 1 year of lactation is only a low-value liquid. Our hypothesis assumes that the milk of mothers who are nursing for more than 1 year or even longer than 2 years is a full-blown milk, containing macronutrients in a composition similar to that of human milk during infancy.

## 2. Materials and Methods

Lactating women were recruited from February 2017 to April 2018 using local parenting groups that communicated via Facebook. One-hundred-and-thirty-seven breastfeeding mothers participated in the study. Age of the women, parity and the route of birth were recorded. Milk collection was overseen at the hospital following the study design during the day shift between 08:00 and 14:00. Samples collected early morning and at night were excluded. A few hours’ time interval provides greater uniformity of samples. The samples were collected from different women. Breast milk was expressed by an electric pump Medela Symphony to sterile cups. Aliquots for analysis (2–3 mL) were taken immediately after complete emptying of the breast and gentle stirring of the whole volume of the expressed milk to minimize the possibility of any preanalytical fault. Milk samples for analysis were divided into four groups according to breastfeeding period: The first group up to 12 months (*n* = 26), the second from 12 to 18 months (*n* = 35), the third from 18 to 24 months (*n* = 41), and the last above 24 months (*n* = 35). For storage, the samples were aliquoted into smaller containers, and frozen at −20 °C.

### Analysis of the Samples

For correct homogenization, each sample was initially heated at 40 °C in an air bath. All analyses were performed in triplicate and each aliquot was homogenized differently. Homogenization using MIRIS Sonicator was performed according to the manufacturer’s instruction. Breast milk macronutrient concentrations—fat, protein, carbohydrate and total solids—were measured simultaneously using a Human Milk Analyzer (HMA) (MIRIS, Uppsala, Sweden) calibrated previously with human milk standards. The obtained results were expressed in g/dL. The equipment requires 2 mL of breast milk and provides the reading of fat, protein, carbohydrate, dry material, true protein, and energy contents. For the technical data, the instrument provided repeatability values of < 0.05%. The HMA uses technology based on the transmission of mid-infrared spectroscopy, designed specifically for the determination of macronutrients in the breast milk. For the calculation of each macronutrient, the instrument uses the amount of radiation absorbed by the different functional groups at specific wavelengths, and it performs an estimate referring to the amount of infrared light absorbed by the distilled water at the same wavelength. The “fat” value provided by the instrument corresponds to the total lipid-soluble fraction of the sample including human milk triglycerides, diglycerides, free fatty acids, phospholipids, and cholesterol. “Crude protein” or “protein” is the protein content based on the total amount of nitrogen (N) in a sample. This means that non-protein nitrogen (NPN) compounds will also be included in this value. “True protein” is corrected for this and represents only the content of actual protein, hence the denotation true. The Miris HMA gives both crude protein and true protein to avoid misunderstandings. Miris HMA uses the factor 6.38 to convert N content to protein content. Total solids and energy are calculated from the Miris HMA measurement results.

The instrument was calibrated by the manufacturer to optimally measure breast milk with its normal biological variation. The habitual use of MIRIS CHECK solution provided by the manufacturer for normal use of the instrument avoids the need for recalibration.

The study received ethical approval from the Ethics Committee of the Medical University of Wrocław (Nr KB–65/2018). All the participating mothers signed their informed written consent.

The statistical analysis was performed with the TIBCO STATISTICA 13.3 software package (StatSoft, Inc., Tulsa, OK, USA). Comparisons between groups were performed by means of the Mann-Whitney U test and the Kruskal-Wallis test. Data are presented as the mean ± SD (standard deviation) and the median with 25th–75th percentiles. The correlations were estimated according to Spearman.

Analysis of variance was performed to determine whether there were statistically significant differences between compared groups of people. In the case of statistically significant differences, an appropriate post-hoc test was used. It made it possible to determine between which groups there were statistically significant differences. The selection was based on the uniformity of variance in the compared groups.

Frequency analysis (N, %) was used in the statistical analysis of the results. A *p*-value lower than 0.05 was regarded as significant.

## 3. Results

The key characteristics of the cohort are detailed in Table 1. All infants and children were exclusively breastfed, but there are no data on the first days after delivery.

### 3.1. Carbohydrates

**Figure 1:**
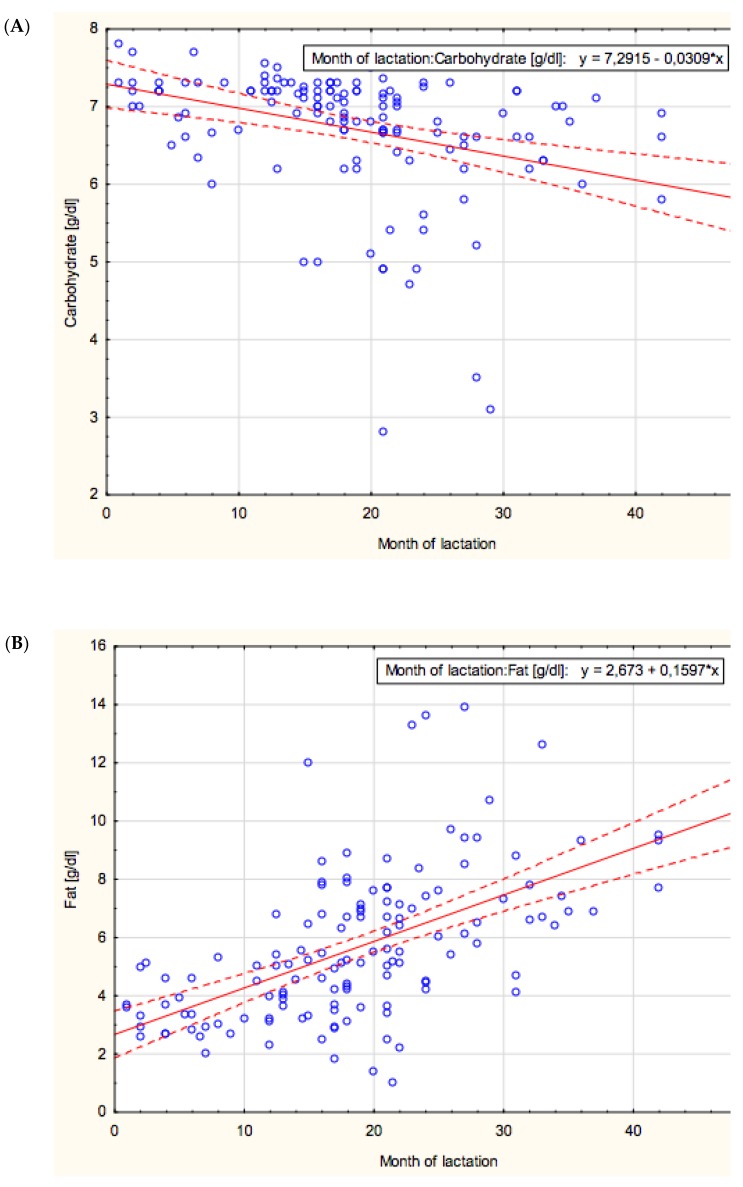

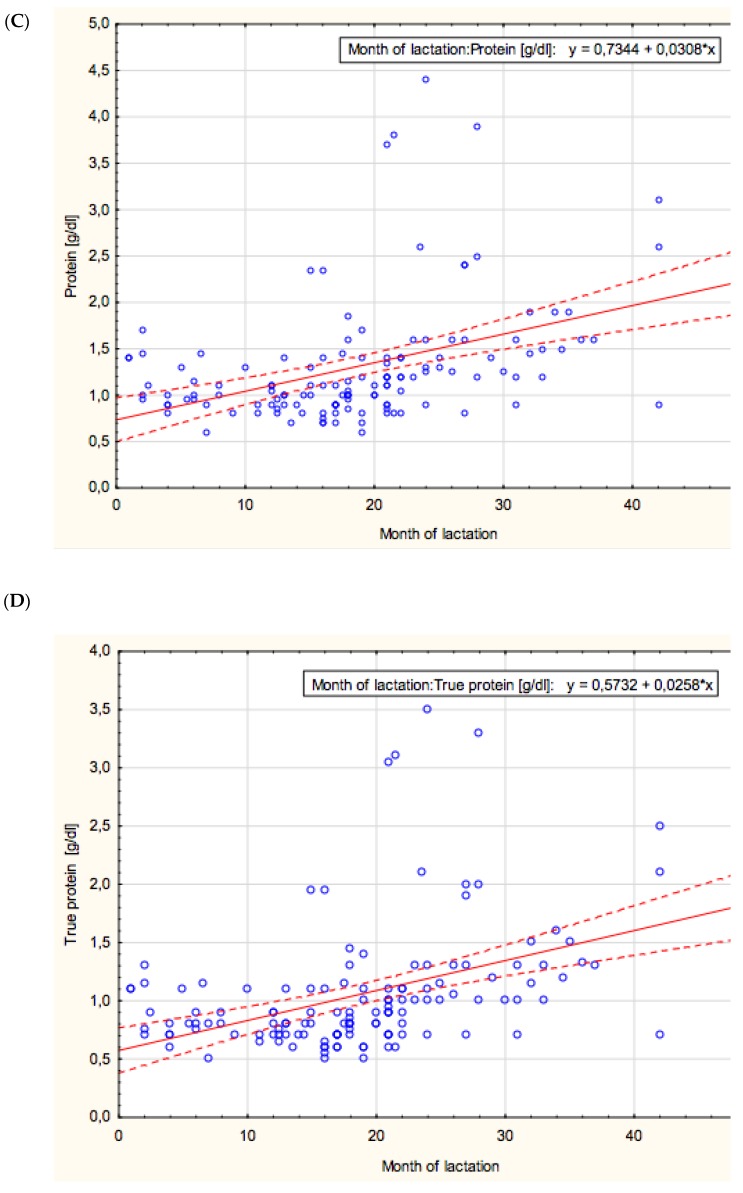

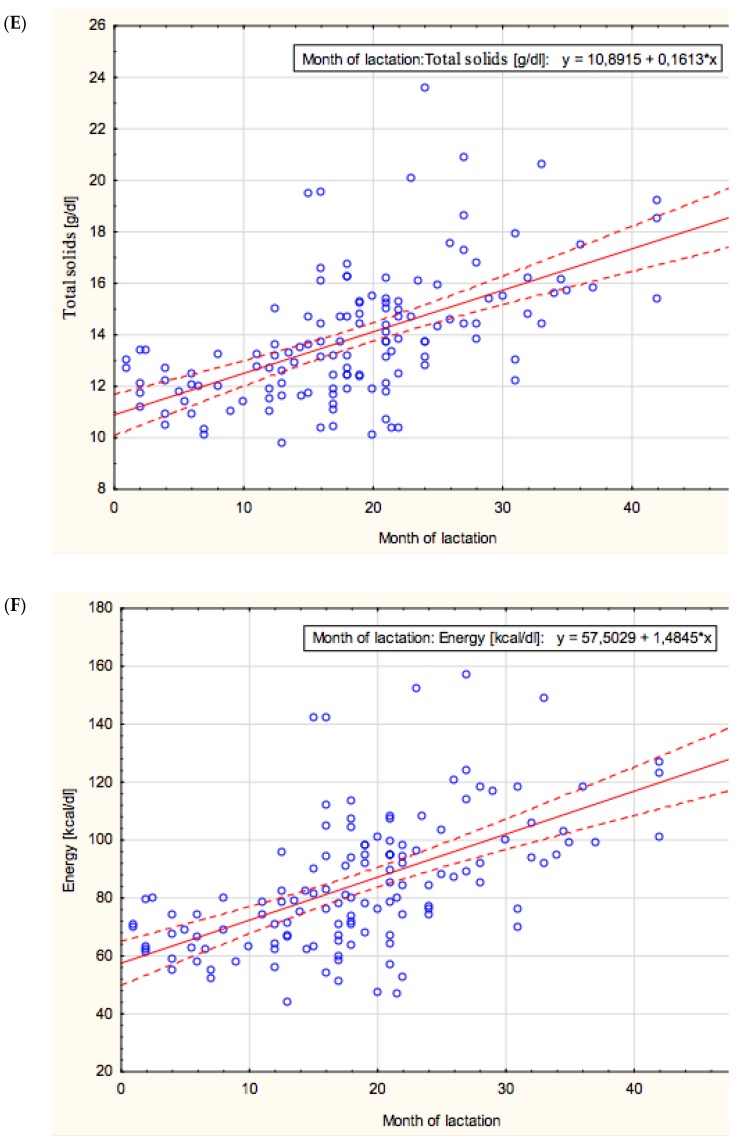
(**A**–**F**). The correlation with the concentration of each macronutrient in mother’s milk and lactation from the 1st to the 48th month. *: multiplied.

The concentration of carbohydrates in mother’s milk showed a negative correlation with lactation from the 1st to the 48th month (*r* = −0.47; *p* < 0.05) (Figure 1A).

The mean value of carbohydrate concentration was stable at a similar level in the groups of 1–12 and 12–18 months of lactation (7.09 ± 0.43 g/dL and 7.03 ± 0.56 g/dL, respectively), then it significantly decreased in the 18–24 months group (6.56 ± 0.93 g/dL; *p* < 0.0002) and remained at a comparable level in the over 24 months group (6.29 ± 0.99 g/dL) (Table 1).

Analysis of variance showed statistically significant differences in carbohydrate content, F (3; 78.2) = 8.19; *p* < 0.001.

### 3.2. Fat

The concentration of fat in mother’s milk showed a positive correlation with lactation from the 1st to the 48th month (*r* = −0.61; *p* < 0.05) (Figure 1B).

The mean value of fat concentration was the lowest in the first analyzed group, namely 1–12 months of lactation, and reached 3.46 ± 0.87 g/dL, and then significantly increased over prolonged lactation to reach 4.91 ± 2.04 g/dL (*p* < 0.002) in the 12–18 months group, 5.77 ± 2.28 g/Dl (*p* < 0.04) in the 18–24 months group and finally 7.95 ± 2.48 g/dL (*p* < 0.0003) in the over 24 months group (Table 1).

Analysis of variance showed statistically significant differences in fat content, F (3; 133) = 3.67; *p* < 0.05.

### 3.3. Protein/True Protein

The concentration of protein and “true protein” in mother’s milk showed a positive correlation with lactation from the 1st to the 48th month (*r* = 0.44; *p* < 0.05 and *r* = 0.45; *p* < 0.05, respectively) (Figure 1C,D).

The mean values of protein and “true protein” concentrations were stable at a similar level in the groups of 1–12 and 12–18 months of lactation (1.08 ± 0.25 g/dL and 1.04 ± 0.38 g/dL, for protein and 0.86 ± 0.2 g/dL and 0.83 ± 0.32 g/dL, for “true protein”, respectively).

In subsequent lactation periods, namely 18–24 and over 24 months of lactation, both protein and “true protein” concentrations significantly increased and reached 1.24 ± 0.64 g/dL (*p* < 0.03) and 1.85 ± 0.87 g/dL (*p* < 0.000005), for protein and 1 ± 0.52 g/dL (*p* < 0.02) and 1.51 ± 0.71 g/dL (*p* < 0.000003), for “true protein”, respectively (Table 1).

Analysis of variance showed statistically significant differences in total protein content, F (3; 50.66) = 10.3, *p* < 0.001, and true protein content, F (3; 51.12) = 10.73, *p* < 0.001.

### 3.4. Total Solids

The total solid’s concentration in mother’s milk showed a positive correlation with lactation from the 1st to the 48th month (*r* = 0.61; *p* < 0.05) (Figure 1E).

The mean value of total solid’s concentration in mother’s milk was the lowest in the first analyzed group, namely 1–12 months of lactation, and reached 11.86 ± 0.95 g/dL, and then significantly increased over prolonged lactation in the group of 12–18 months of lactation (13.19 ± 2.21 g/dL; *p* < 0.007) and remained at a similar level in the group of 18–24 months of lactation (13.82 ± 2 g/dL). Finally, the concentration of total solids increased significantly in the group over 24 months of lactation and obtained the highest value of 16.28 ± 2.59 g/dL (*p* < 0.00003) (Table 1).

Analysis of variance showed statistically significant differences in total solids content, F (3; 94.59) = 25.82; *p* < 0.001.

### 3.5. Energy/Calories

The energy/calories of mother’s milk showed a positive correlatio7n with lactation from the 1st to the 48th month (*r* = 0.61; *p* < 0.05) (Figure 1F).

The mean value of energy in mother’s milk was the lowest in the first analyzed group, namely 1–12 months of lactation, and reached 65.76 ± 7.92 kcal/dL and then significantly increased over prolonged lactation and reached 78.34 ± 21.72 kcal/dL (*p* < 0.006) in the 12–18 group, 85.78 ± 20.07 kcal/dL (*p* < 0.04) in the 18–24 months group and finally 106.5 ± 23.46 kcal/dL (*p* < 0.0002) in the over 24 months group (Table 2).

Analysis of variance showed statistically significant differences in calorie content, F (3; 98.78) = 24.4; *p* < 0.001.

### 3.6. Correlation among Macronutrients in Prolonged Lactation

Correlations among macronutrients of mother’s milk over prolonged lactation from the 1st to the 48th month are summarized in Table 3.

The concentration of carbohydrates in milk showed a statistically significant negative correlation with the concentration of fat (*r* = −0.56), protein (*r* = −0.46), “true protein” (*r* = −0.47) as well as with the concentration of total solids (*r* = −0.45) and energy (*r* = −0.53). Moreover, very strong positive correlations between fat and both total solids (*r* = 0.95) and energy (*r* = 0.98) were found, and weak but statistically significant correlations between fat and protein (*r* = 0.35) and true protein (*r* = 0.36) were observed. Additionally, over prolonged lactation from the 1st to the 48th month the concentration of both protein and “true protein” positively correlated with the concentration of total solids (*r* = 0.44) and energy (*r* = 0.40).

Interestingly, during the analyzed period of prolonged lactation, namely over 24 months (from the 25th to the 48th month of lactation), neither the concentration of any of the macronutrients nor the energy content correlated with the progression of lactation (Table 4). In contrast, similarly to the overall analyzed period, namely from the 1st to the 48th month of lactation, the concentrations of macronutrients correlated with each other, but there was no correlation between carbohydrates and proteins.

### 3.7. Macronutrient and Energy Content in Breast Milk in Relation to the Amount of Feeding

The correlations between the macronutrients of mother’s milk with the amount of feeding during three stages of prolonged lactation are summarized in Table 4.

**Figure 2:**
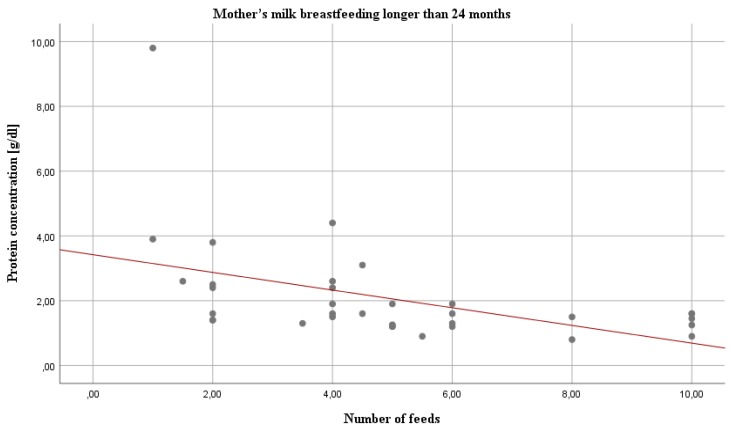
Relation between the concentration of protein and the amount of feeding in mother’s milk over 24 months of lactation. The number of feedings in the analyzed group ranged from 1 to 10 per day.

Statistical analysis revealed several statistically significant correlations in two out of three groups. Namely, in the 18–24 months group, the amount of feeding was positively correlated with the carbohydrate content (*r* = 0.31; *p* < 0.05) (Table 5). In contrast, in the over 24 months group, all analyzed macronutrients were positively (carbohydrate, *r* = 0.51) or negatively (fat, *r* = −0.36; protein and true protein, *r*= −0.58 and *r*= −0.58, respectively) (Figure 2) correlated with the amount of feeding.

### 3.8. Macronutrient and Energy Content in Breast Milk in Relation to Parity

**Figure 3:**
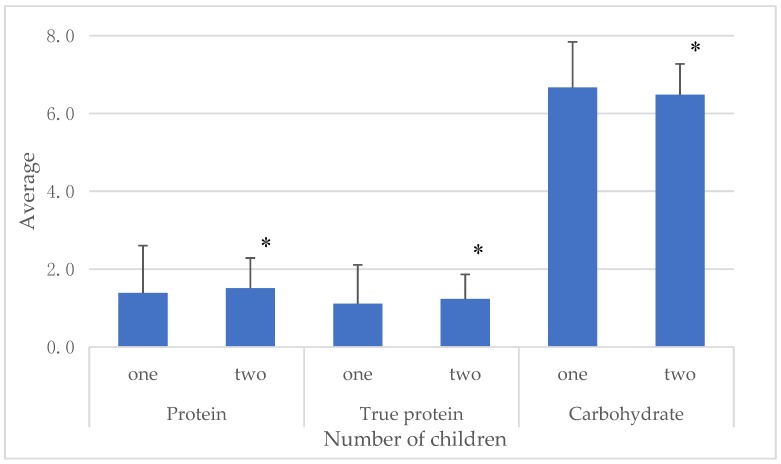
Concentrations of total protein, true protein and carbohydrates in the group of women with one or two children. * *p* < 0.05.

The statistically significant differences in macronutrient concentrations, namely protein (U = 963; *p* < 0.05), true protein (U = 920; *p* < 0.05) and carbohydrates (U = 983.5; *p* < 0.05), during prolonged lactation among mothers having one or two children are shown in Figure 3. In the group of women with one child, the levels of total and true protein in milk were statistically significantly lower compared to women with two children. In contrast, the concentration of carbohydrates was higher in milk of mothers having the first child.

## 4. Discussion

Analysis of the composition of long-breastfeeding mother’s milk allows one to assess the nutritional value. Human milk is a source of proteins, carbohydrates and fatty acids—essential macronutrients. From a nutritional perspective, infancy is a critical and vulnerable period. The macronutrient composition of human milk varies among mothers and across lactation. The composition of mature, term milk is estimated to be approximately 0.9 to 1.2 g/dL for protein, 3.2 to 3.6 g/dL for fat, and 6.7 to 7.8 g/dL in the first year of lactation. Energy estimates range from 65 to 70 kcal/dL, and are highly correlated with the fat content of human milk [14]. We demonstrated that macronutrient contents expressed by mothers breastfeeding for longer than 1 year, namely fats, proteins and energy content, were increased significantly, compared with HM expressed by women breastfeeding up to 12 months. The same results have been show by Lubetzky et al., in 2012 [34]. The authors showed that HM produced above 1 year of lactation is extraordinarily rich in fat and has a higher energy content than human milk produced during the first 6 months of lactation [34].

In the group of mothers breastfeeding for more than 2 years, the concentrations of fat and protein were the highest in comparison to other analyzed groups. This fact is probably related to the adaptation of milk composition to the increased energy demand of the intensively growing child. Moreover, no significant correlations among macronutrients and month of lactation in the group of mothers breastfeeding longer than 24 months was observed. The concentrations of fat and protein increase over prolonged lactation but only up to 2 years, after which the concentrations remain at a stable level, although it is correlated with the amount of feeding. In contrast, the concentration of carbohydrates over lactation is significantly decreased. As reported by Nommsen and coworkers (1991), nearly 2.5-fold higher concentration of fat in human milk is inversely related to the milk volume intake of infants [35]. These results show that the source of calories in breast milk for older children is mainly fat, while carbohydrates play a greater role in infant nutrition in the early stage of life. Higher concentration of fats and lower concentration of carbohydrates may be associated with lower gains in adiposity and BMI [36]. On the other hand, there was no impact of BMI on macronutrient content of human colostrum [37], however, this issue requires further research. Also, the duration of breastfeeding does not affect lipid profiles in young adults as has been shown by Hayosh et al., 2015. These results undermine the Hertfordshire study which suggested that the age of weaning and methods of infant feeding may influence adult serum low-density lipoprotein cholesterol (LDL-C) and mortality from ischemic heart disease in men [38]. The altered composition of breast milk, and the downstream effects this may have on subsequent adult health, are of great interest in regard to the metabolic programming during this early period.

During prolonged lactation, the contribution of breast milk to the infant diet might be significant, from an energy intake standpoint. Indeed, a reduction in the volume of milk consumed by a child who is also eating solid foods might well be counterbalanced by the increase in energy level [30,39,40]. A limitation of this study was that it was not possible to measure the volumes consumed by each infant, because of difficulties with expressed milk from mothers breastfeeding for a long time. Only specialist equipment in the hospital gave the opportunity to express the milk. The fatty acid profile of human milk is altered by diet, but a bigger difference was observed in the group of mothers breastfeeding longer than 24 months. It may be related to the fact that as the length of lactation increases, the body’s resources are exhausted, and the diet becomes more important.

It should be clearly emphasized that, at the present time, the official policy of the American Academy of Pediatrics is not to put any limit on the duration of lactation. Moreover, review of biological versus cultural aspects of weaning suggested that, from an anthropologic standpoint based on primates studies, “breastfeeding a child for 2.5 to 7 years is normal for our species” [30,41]. The same changes as shown in this study in macronutrient composition were observed during weaning. Significant changes in milk protein and lactose concentrations were observed only when milk volume fell below 400 mL/day; more than one feed per day was necessary to maintain milk secretion. Differences between the relation of milk volume and composition during lactogenesis and weaning suggest that volume is differently regulated in the two periods [12]. It can therefore be concluded that long-term breastfeeding is a natural way to slow termination of feeding and lactation. Due to greater awareness of nutrition in the first months and years of a child’s life, long-term breastfeeding is more common among highly educated, better-off mothers who are more health conscious [42]. The protection, promotion, and support of breastfeeding are essential for maternal and child health. Findings from epidemiological and biological studies demonstrated that the decision not to breastfeed has major long-term effects on the health, development and nutrition of the child. Health of the mother is also strongly correlated with the duration of lactation, namely breastfeeding continued up to the age of 2 years protect against breast cancer. Protection against mortality and morbidity from infectious diseases extends well into the second year of life, and breastfeeding prevents half of the deaths caused by infections in children aged 6–23 months. Ethnographical research shows that the total duration of breastfeeding ranges between 2 and 4 years in most traditional societies [43,44,45].

## Figures and Tables

**Table 1:** Characteristics of the Study Population.

Outcome and Exposure Variables	Breastfeeding < 12 Months *n* = 26 (% (n/N))	Breastfeeding > 12 Months *n* = 111 (% (n/N))
**Maternal age**		
25–29	42.3 (11/26)	33.3 (37/111)
30–34	46.2 (12/26)	43.2 (48/111)
≥35	11.5 (3/26)	23.5 (26/111)
**Race/ethnicity**		
Caucasian	100(26/26)	100 (111/111)
**Socioeconomic status and education**		
Secondary education	15 (4/26)	20 (22/111)
High education	85 (22/26)	80 (89/111)
**Number of children**		
1	77 (20/26)	63.3 (69/109)
2	23 (6/26)	25.7 (38/109)
3	(0/26)	2 (2/109)
**Birth weight**		
Appropriate for gestational age (AGA)	100 (26/26)	98.2 (109/111)
**Gestational age**		
34–37	11.5 (3/26)	1.8 (2/111)
>37	88.5 (23/26)	98.2 (109/111)
**Medicines taken during lactation**		
No medications	65.5 (17/26)	68.5 (76/111)
Thyroxine	23 (6/26)	19 (21/111)
Others	11.5 (3/26)	12.5 (14/111)
**Maternal diet during lactation**		
Vegan/vegetarian	0 (0/26)	10 (11/111)
Dairy-free diet	19.2 (5/26)	9 (10/111)
Gluten-free diet	0 (0/26)	2.7 (3/111)
**Complementary foods introduction**		
Over 6 month of life	NA	90 (100/111)

NA—not assessed.

**Table 2:** Macronutrient and energy content in breast milk in prolonged lactation.

Breast Milk Macronutrient Content	Group			
	1–12 Month *n* = 25	12–18 Month *n* = 35	18–24 Month *n* = 41	>24 Month *n* = 34
**Carbohydrate** [g/dL]	7.09 ± 0.43	7.03 ± 0.56	6.56 ± 0.93 **	6.29 ± 0.99
	7.2	7.20	6.8	6.6
	6.86–7.30	7.00–7.30	6.40–7.10	5.8–6.9
			*p* < 0.0002	
**Fat** [g/dL]	3.46 ± 0.87	4.91 ± 2.04 *	5.77 ± 2.28 **	7.95 ± 2.48 ***
	3.30	4.60	5.60	7.50
	2.80–3.90	3.50–5.55	4.40–7.10	6.40–9.40
		*p* < 0.002	*p* < 0.04	*p* < 0.0003
**Protein** [g/dL]	1.08 ± 0.25	1.04 ± 0.38	1.24 ± 0.64 **	1.85 ± 0.87 ***
	1.00	0.90	1.1	1.6
	0.90–1.30	0.80–1.10	0.90–1.35	1.25–2.4
			*p* < 0.03	*p* < 0.000005
**True protein** [g/dL]	0.86 ± 0.2	0.83 ± 0.32	1 ± 0.52 **	1.51 ± 0.71 ***
	0.80	0.70	0.90	1.30
	0.70–1.10	0.65–0.90	0.70–1.00	1.00–1.90
			*p* < 0.02	*p* < 0.000003
**Total solids** [g/dL]	11.86 ± 0.95	13.19 ± 2.21 *	13.82 ± 2	16.28 ± 2.59 ***
	12.0	13.1	13.80	15.75
	11.20–12.70	11.65–13.70	12.40–15.20	14.40–17.55
		*p* < 0.007		*p* < 0.00003
**Energy** [kcal/dL]	65.76 ± 7.92	78.34 ± 21.72 *	85.78 ± 20.07 **	106.5 ± 23.46 ***
	63.0	76.0	89.5	102.0
	61–71	64–83	73.5–98	89–118
		*p* < 0.006	*p* < 0.04	*p* < 0.0002

Values are given as the mean ± SD, median and 25th–75th percentiles. The Mann-Whitney *U*-test was used for statistical calculations, and a *p*-value lower than 0.05 was regarded as significant. Significantly different from the milk group of: * 1–12 months of lactation, ** 12–18 months of lactation, *** 18–24 months of lactation.

**Table 3:** Correlations among macronutrients over prolonged lactation from the 1st to the 48th month.

Correlation Coefficient *r* Value *							
	Month of Lactation	Carbohydrate	Fat	Protein	True Protein	Total Solids	Energy
Carbohydrate	–0.47	---	–0.56	–0.46	–0.47	–0.45	–0.53
Fat	0.61		---	0.35	0.36	0.95	0.98
Protein	0.44			---	0.98	0.44	0.40
True protein	0.45				---	0.44	0.40
Total solids	0.61					---	0.99
Energy	0.61						---

The values of r calculated according to the Spearman method correspond to the correlation between the concentrations of macronutrients over prolonged lactation from the 1st to the 48th month. * All *r* values are statistically significant with *p* < 0.05.

**Table 4:** Correlations among macronutrients during lactation over 24 months.

Correlation Coefficient *r* Value *							
	Month of Lactation	Carbohydrate	Fat	Protein	True Protein	Total Solids	Energy
Carbohydrate	NS	---	−0.60	NS	−0.36	−0.35	−0.47
Fat	NS		---	0.35	0.42	0.89	0.96
Protein	NS			---	0.98	0.53	0.46
True protein	NS				---	0.57	0.51
Total solids	NS					---	0.97
Energy	NS						---

The values of r calculated according to the Spearman method correspond to the correlation between the concentrations of macronutrients over prolonged lactation from the 25th to the 48th month. * All *r* values are statistically significant with *p* < 0.05; NS not significant.

**Table 5:** The relationship between the number of feedings and macronutrient concentration in breast milk during prolonged lactation.

Macronutrient	Number of Feedings *		
	12–18 Month	18–24 Month	>24 Month
Fat	−0.02	0	−0.36 *
Protein	−0.08	−0.27	−0.58 ***
Carbohydrate	0.14	0.31 *	0.51 **
Total solids	−0.02	−0.05	−0.28
Energy	−0.06	−0.08	−0.34 *
True protein	−0.14	−0.29	−0.58 ***

* The number of feedings in the analyzed groups ranged from 1 to 10 per day. The values of *r* calculated according to the Spearman method correspond to the correlation between the number of feedings and macronutrient concentration in breast milk during three periods of prolonged lactation from the 12th to the 48th month. *r* values are statistically significant with * *p* < 0.05; ** *p* < 0.01; *** *p* < 0.001.
